# The zoonotic pathogen *Leptospira interrogans* mitigates environmental stress through cyclic-di-GMP-controlled biofilm production

**DOI:** 10.1038/s41522-020-0134-1

**Published:** 2020-06-12

**Authors:** Roman Thibeaux, Marie-Estelle Soupé-Gilbert, Malia Kainiu, Dominique Girault, Emilie Bierque, Julien Fernandes, Heike Bähre, Anthony Douyère, Nicolas Eskenazi, Joëlle Vinh, Mathieu Picardeau, Cyrille Goarant

**Affiliations:** 1 Institut Pasteur de Nouvelle-Calédonie, Unité de Recherche et d’Expertise sur la Leptospirose, Noumea, New Caledonia; 2 Institut Pasteur, UTechS PBI, Centre de Ressources et Recherches Technologiques (C2RT), Paris, France; 3 Hannover Medical School, Research Core Unit Metabolomics, Hannover, Germany; 4 Université de la Nouvelle-Calédonie, Institut des Sciences Exactes et Appliquées, Plateau MET/MEB, Noumea, New Caledonia; 5 École Supérieure de Physique et de Chimie Industrielles de la ville de Paris, Spectrométrie de Masse Biologique et Protéomique, CNRS, Université Paris-Sciences-et-Lettres, Paris, France; 6 Institut Pasteur, Unité Biologie des Spirochètes, Paris, France

**Keywords:** Biofilms, Environmental microbiology

## Abstract

The zoonotic bacterium *Leptospira interrogans* is the aetiological agent of leptospirosis, a re-emerging infectious disease that is a growing public health concern. Most human cases of leptospirosis result from environmental infection. Biofilm formation and its contribution to the persistence of virulent leptospires in the environment or in the host have scarcely been addressed. Here, we examined spatial and time-domain changes in biofilm production by *L. interrogans*. Our observations showed that biofilm formation in *L. interrogans* is a highly dynamic process and leads to a polarized architecture. We notably found that the biofilm matrix is composed of extracellular DNA, which enhances the biofilm’s cohesiveness. By studying *L. interrogans* mutants with defective diguanylate cyclase and phosphodiesterase genes, we show that biofilm production is regulated by intracellular levels of bis-(3′–5′)-cyclic dimeric guanosine monophosphate (c-di-GMP) and underpins the bacterium’s ability to withstand a wide variety of simulated environmental stresses. Our present results show how the c-di-GMP pathway regulates biofilm formation by *L. interrogans*, provide insights into the environmental persistence of *L. interrogans* and, more generally, highlight leptospirosis as an environment-borne threat to human health.

## Introduction

Pathogenic species of bacteria from the genus *Leptospira* cause leptospirosis, a zoonosis that has been observed worldwide but is especially prevalent in tropical low-income countries^1^. The disease affects 1.03 million humans annually, and kills nearly 60,000^2^. Pathogenic leptospires persist in the renal tubules of chronically infected reservoir animals, and are excreted in the urine^3^—thus contaminating the environment. Leptospires can survive in the environment for months^4,5^. Contact with a contaminated environment is the predominant cause of transmission to humans^6^. Pathogenic *Leptospira* are able to produce a biofilm in vitro^7,8^ and the presence of pathogenic *Leptospira* within a biofilm in the environment has been reported—suggesting that this structure has an important role in *Leptospira*’s ability to survive *in natura*^4,9^ and in water distribution systems^10,11^. Studies of reservoir mammals have highlighted the presence of bacterial aggregates within the lumen of proximal renal tubules^12,13^. These aggregates share some morphological features with biofilms, which might explain why *Leptospira* are so persistent and why antibiotic treatments fail to clear the bacteria from the kidneys^14^.

Biofilms are multicellular aggregates embedded in a three-dimensional self-produced matrix that confers protection against adverse conditions such as desiccation, osmotic shock, and exposure to some types of toxic compounds, UV radiation and predators^15–20^. The matrix formed by bacteria is held together by interconnecting compounds, such as self‐produced polysaccharides, proteins, extracellular DNA (exDNA) and cell lysis products, and material from the surrounding environment^21^.

*Leptospira*’s ability to remain viable and infectious in various environments (such as the soil, water, and mammalian hosts) suggests that these bacteria have fast-acting, sensitive regulatory systems that enable them to adapt to various environmental challenges. In many biofilm-producing bacterial species studied to date, biofilm formation is regulated by the signalling molecule bis-(3′-5′)-cyclic dimeric guanosine monophosphate (c-di-GMP)^22–24^. C-di-GMP is produced by diguanylate cyclases (DGCs) and degraded by phosphodiesterases (PDEs)^25,26^. Production of this intracellular signalling molecule is low in motile cells whereas its elevation in the cytoplasm triggers biofilm formation^27,28^. Our understanding of c-di-GMP turnover in *Leptospira* is limited^29,30^. An analysis of the *L. interrogans* genome found 25 proteins predicted to be involved in c-di-GMP metabolism^31^. Some of these enzymes were found to be active in vivo^30^.

Although persistent forms have not been described to date, we hypothesized that *L. interrogans*’ biofilm helps virulent leptospires to endure unfavourable environments. We, therefore, sought to better understand the role of c-di-GMP in the formation, organization, composition, role and regulation of biofilms in this bacterium. This study increased our understanding of how *L. interrogans’* biofilm is formed and organized. In particular, we revealed the critical role of exDNA in biofilm cohesion. By studying mutants with impairments in the c-di-GMP pathway, we illustrated the key role of intracellular c-di-GMP in controlling biofilm formation and motility in *L. interrogans*. We also showed that biofilm production confers resistance to simulated environmental stresses but does not seem to promote virulence or chronic infectivity.

## Results

### Biofilm formation is a collective and dynamic process

**Fig. 1:**
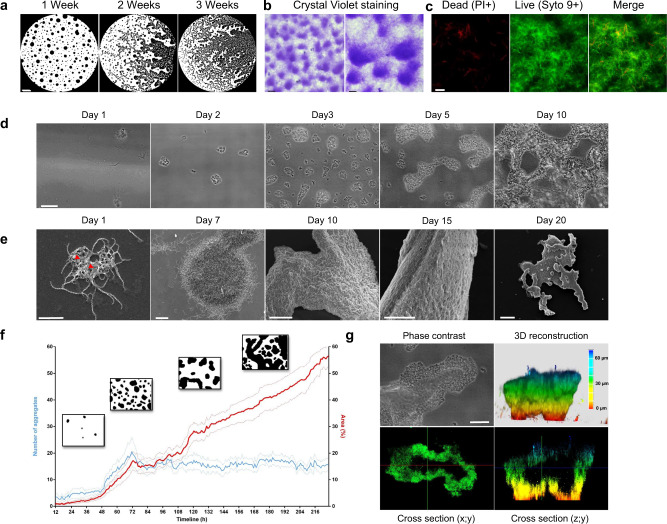
*Leptospira* biofilm formation. **a** Phase contrast images of *Leptospira* biofilm at 1, 2 and 3 weeks post-inoculation. Scale bar: 500 µm. **b** Phase contrast images of Crystal Violet-stained biofilms Scale bars: 100 µm (left); 50 µm (right). **c** CLSM images of *Leptospira* biofilm stained with Propidium Iodide (PI, left panel) and Syto 9 (central panel). Merged image is shown in the right panel. Scale bar: 10 µm. **d** Phase contrast images of *Leptospira* biofilm formation at 1, 2, 3, 5 and 10 days post-inoculation. Scale bar: 50 µm. **e** SEM images of *Leptospira* biofilm formation at 1, 7, 10, 15 and 20 days post-inoculation. Red arrowheads show ECM deposit. Scale bars: 5 µm (day 1); 10 µm (day 7); 50 µm (days 10 and 15); 100 µm (day 20). **f** Quantitative phase-contrast image analysis of the number of biofilm aggregates (blue curve) and the biofilm area (red curve) over time between 12 and 216 h post-inoculation. The dotty curves represent the standard error of the mean. Insets show binarized images representative of biofilm aspect at 36, 72, 132 and 180 h post-inoculation. **g** Correlative phase contrast-CLSM image of biofilm stained with CFDA-SE Cell tracker performed on a native non-fixed biofilm. Phase contrast image (upper left panel), 3D-reconstruction (upper right panel), XY cross-section (lower left panel) and ZY cross-section (lower right panel) are shown. Scale bar: 20 µm.

We first sought to describe biofilm formation by *L. interrogans*. To this end, we applied a combination of phase-contrast microscopy, time-lapse imaging, confocal laser scanning microscopy (CLSM), and scanning electron microscopy (SEM) (Fig. 1). The phase-contrast microscopy data (Fig. 1a) indicated that the *Leptospira* biofilm changes its shape significantly over time through an expansion process. These bacterial aggregates’ sugar moieties were stained with Crystal violet (Fig. 1b). Additional live/dead staining of the biofilm aggregates (Fig. 1c) showed that most of the bacteria in the biofilms were alive. The biofilm’s shape varied markedly from one *Leptospira* species to another, with dotted, branching and reticulated patterns (Supplementary Fig. 1A).

Our time-lapse microscopy and SEM images revealed the whole sequence of biofilm formation (Fig. 1d, e). Motile planktonic *L. interrogans* cells were observed shortly after bacteria had been seeded in the glass slide chamber. Small dots of multicellular structures then appeared over the first 72 h of culture (Fig. 1d–f). Live imaging showed that microcolonies originated from the aggregation of planktonic bacteria which were steadily depleted from the supernatant (Supplementary Fig. 1B). These multicellular structures were not anchored to the culture dish but remained motile for up to seven days, and moved together on the substrate surface (Supplementary Movie 1). As more and more multicellular structures appeared over time, we observed collisions and interconnections—leading to massive aggregates that expanded across the surface of the microscope slide (Fig. 1e, f) and, ultimately, stopped moving due to a lack of space. Supplementary Fig. 1C shows a coalescence event that occurred on day three and led to a decrease in the number of aggregates. Extracellular matrix (ECM) deposits were visible under the SEM as early as day 1 (Fig. 1e). After a week, the mature biofilms had grown perpendicularly to the substrate (Fig. 1e, g) and were more than 50 µm thick. Our CLSM experiments systematically highlighted the presence of a void within some aggregates (Fig. 1g) formed by a particular migration of the bacteria; after spreading out, the aggregate contracted to create the void. The full sequence of events is shown in Supplementary Fig. 1D and Supplementary Movie 2.

### The biofilm’s architecture is highly polarized

**Fig. 2:**
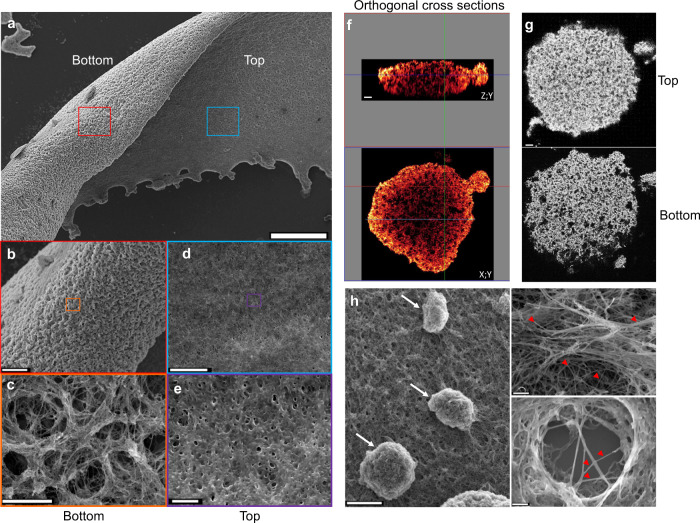
*Leptospira* biofilm architecture and organization. **a** SEM images of the apical and basal sides of *Leptospira* biofilm after 3 weeks of static culture Scale bar: 200 µm. **b**, **c** Higher magnification of the biofilm basal side showing large interconnected channels. Scale bars: 50 and 5 µm. **d**, **e** Higher magnification of the biofilm apical side showing a flat surface of bacteria enmeshed within an amorphous ECM Scale bars: 10 and 2 µm. **f** ZY (upper panel) and XY (lower panel) orthogonal cross-sections of CLSM images of bacteria stained with CFDA/SE Green CellTracker, showing the foam-like architecture of *Leptospira* biofilm. Scale bar: 10 µm. **g** CLSM images at the top (upper panel) and the bottom (lower panel) sides of a *Leptospira* biofilm illustrating the density gradient along the baso-apical polarity axis. Scale bar: 10 µm. **h** SEM images of a mature biofilm showing budding-like structures (left panel, arrows), and branching extracellular matrix filaments (right panels, red arrowheads). Scale bars: 20 µm (left); 2 µm (upper right); 1 µm (lower right).

A thorough SEM and CLSM analysis of the mature biofilm (Fig. 2) highlighted a number of characteristic structural features. The basal (substrate) side of the biofilm had a rough texture (Fig. 2a, b), and large channels (>five µm in diameter) were visible. The spiral-shaped bacteria could be clearly seen because little or no ECM was present (Fig. 2c). Given the porous nature of the basal side of the biofilm, the entire structure was anchored to the substrate by a small contact area. In contrast, the apical (exposed) side of the biofilm was flat and smooth (Fig. 2d). High-magnification imaging revealed tiny pores (diameter < 1 µm) and bacteria enmeshed within a thick matrix. An analysis of three-dimensional sections of the mature biofilm (Fig. 2f) confirmed the porous, foam-like nature of the structure. The channels that permeated the entire biofilm were wider on the basal side than on the apical side (Fig. 2g). The mature biofilm contained striking phenotypic features, such as mushroom-like structures and branching extracellular polymeric filaments (Fig. 2h).

### Extracellular DNA has a structural role in the *Leptospira* biofilm

**Fig. 3:**
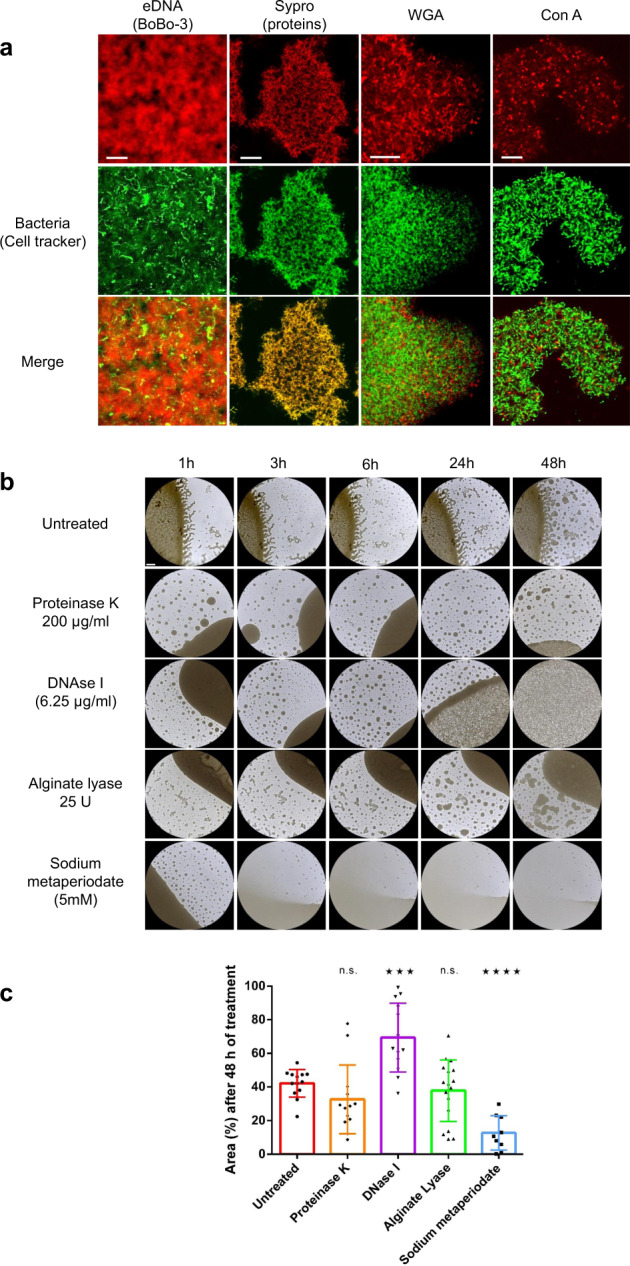
Composition of the biofilm matrix. **a** CLSM micrographs of biofilms stained with BOBO-3 (exDNA, Scale bar: 10 µm), Sypro Ruby (proteins, Scale bar: 20 µm), WGA (N-acetylglucosamine or sialic acid residues, Scale bar: 10 µm) and ConA (red/α-D-glucosyl or α-D-mannosyl residues, Scale bar: 10 µm), red, upper line. Bacteria were stained with a CFDA-SE cell-tracker (green, middle line). Merged images are shown in the lower line. **b** Phase contrast images of biofilms left untreated or treated for 1, 3, 6, 24 and 48 h with proteinase K (1 µg/mL), DNase I (6.25 µg/mL), alginate lyase (125 U/mL) and sodium metaperiodate (5 mM). Scale bar: 500 µm. **c** Surface based quantification of the area occupied by the biofilm after 48 h of exposure to proteinase K (orange), DNase I (purple), Alginate Lyase (green) and sodium metaperiodate (blue). **** indicates a *p* value < 0.0001; *** indicates a *p* value < 0.001; ns indicates a non-significant *p* value (>0.05) in two-tailed unpaired Mann–Whitney test compared to the untreated condition. Error bars indicate standard deviation.

We used fluorescent probes to assess the mature biofilm’s chemical composition (Fig. 3a and Supplementary Fig. 2A). Extracellular DNA was abundant within the mature biofilm. No proteins, other than those closely associated to the bacterial body and surface were detected in the biofilm, suggesting that proteins are not the main component of the ECM. Specific wheat germ agglutinin staining of N-acetyl-d-glucosamine and N-acetylneuraminic acid confirmed that these residues were present in the matrix. Additional concanavalin A staining revealed the presence of x-mannopyranosyl and x-glucopyranosyl moieties within the matrix. We further explored the matrix’s composition by using enzymatic treatments (Fig. 3b, c and Supplementary Fig. 2B). Exposure to proteinase K or alginate lyase did not dissociate the biofilm structure. Biofilm quantification after 48 h of exposure to both enzymes did not reveal any differences as compared to the untreated condition. Conversely, DNase disrupted the biofilm after 24 h, with a significant loosening of the biofilm’s overall structure. This effect was reflected by an important increase in the surface area occupied by the biofilm (Fig. 3c). Sodium periodate (used to open glycosidic rings by cutting a vicinal diol’s carbon-carbon bond) dissolved the biofilm in less than 3 h leading to a significant decrease of the quantified biofilm surface (Fig. 3c).

### The intracellular level of c-di-GMP regulates biofilm production

**Fig. 4:**
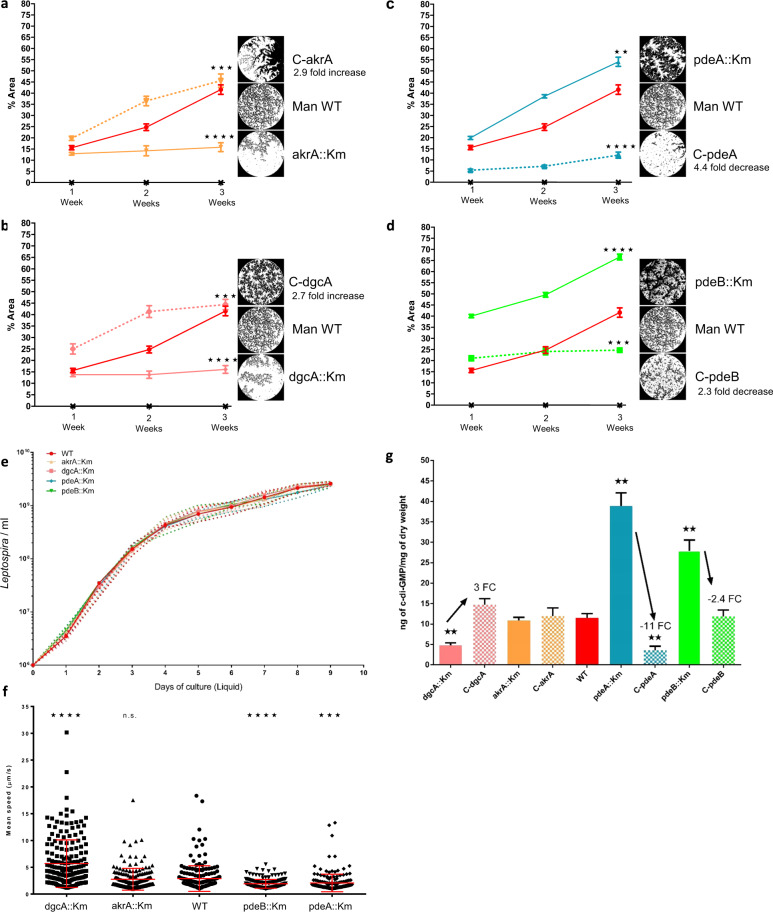
C-di-GMP intracellular level regulates *Leptospira* motility and biofilm production. Mutant akrA::Km is shown in orange, dgcA::Km in pink, pdeA::Km in blue and pdeB::Km in green. Error bars represent the standard error of the mean. **a** Quantification of biofilm production at 1, 2 and 3 weeks post-inoculation for WT Manilae (red), Manilae mutants (solid line) and complemented mutants (dotty line): C-akrA (**a**), C-dgcA (**b**), C-pdeA (**c**), C-pdeB (**d**). Insets show a representative phase contrast image of the biofilm growth for the different strains. **e** Proliferation kinetics in liquid EMJH of WT and mutant strains represented as the concentration of *Leptospira* per mL over a 9-day period. **f** Mean displacement speed in µm.s^−1^ measured in vitro in liquid EMJH for WT and mutant Manilae strains. *n* = 190 cells per condition. **g** Quantification of intracellular c-di-GMP levels in WT, mutant and complemented mutant strains. **** indicates a *p* value < 0.0001; *** indicates a *p* value < 0.001; ** indicates a *p* value < 0.01 in two-tailed unpaired Mann–Whitney test compared to the WT strain.

We assessed the ability to form a biofilm in 15 *L. interrogans* transposon insertion mutants with gene knockouts in the c-di-GMP pathway or for surface adhesins/sugar metabolism enzymes. Based on their biofilm production phenotype, four mutants were selected for further study (Fig. 4a–d). Three transposon insertions occurred within the c-di-GMP synthesis pathway, namely in the mutants dgcA::Km (LMANV2_v2_150005; GGDEF-only; DGC, c-di-GMP synthesis), pdeA::Km (LMANV2_v2_270021; EAL-only; PDE, c-di-GMP degradation), and pdeB::Km (LMANV2_v2_90001; EAL-only; PDE, c-di-GMP degradation). The fourth mutant (akrA::Km) carried a mutation in an aldoketoreductase-encoding gene (LMANV2_v2_50029) that is reportedly over-expressed and involved in biofilm production in *Staphylococcus aureus* and *Vibrio cholerae*^32,33^. After 3 weeks of culture, the proportion of biofilm surface coverage was 15.8% for akrA::Km, 16.0% for dgcA::Km, and 44.6% for the wild-type strain (Fig. 4a, b). Conversely, pdeA::Km and pdeB::Km displayed abnormally high levels of biofilm production, with surface coverages of 54.2% and 57.2%, respectively (Fig. 4c, d). The mutants’ phenotypes were rescued by complementation with the WT gene. The complemented C-akrA and C-dgcA strains completely recovered their ability to produce a biofilm, with 2.9- and 2.7-fold increases in the surface coverage, respectively, (Fig. 4a, b). The C-pdeA and C-pdeB strains also changed their phenotype (Fig. 4c, d) but the proportion of biofilm surface coverage fell to a value below that of the WT parental strain, with a 4.4- and 2.3-fold decrease in biofilm production respectively. Trans-complementation may not restore gene expression to WT levels, even when a low-copy-number plasmid is used for complementation.

We assessed changes in *Leptospira*’s proliferative and motile capacities. The four mutant strains exhibited the same growth kinetics as the WT strain, and did not display any impairments in proliferation (Fig. 4e). However, we observed discrepancies in velocity (Fig. 4f and Supplementary Table 1). The velocity was significantly higher in dgcA::Km than in the WT. In contrast, pdeA::Km and pdeB::Km were less motile than the WT. Given that akrA::Km was not affected in the c-di-GMP pathway, this strain was used as a control for a c-di-GMP-independent defect in biofilm production; its velocity was similar to that of the WT. We next assessed the impact of mutations in DGCs (using the dgcA::Km strain) or PDEs (using pdeA::Km and pdeB::Km) on intracellular c-di-GMP concentrations (Fig. 4g and Supplementary Table 2). The level was significantly lower in dgcA::Km than in the WT; this was expected because the corresponding gene product is involved in c-di-GMP synthesis. A 3-fold increase was observed after complementation with the gene encoding a functional DGC. Conversely, as expected, the intracellular c-di-GMP levels were greatly elevated in pdeA::Km and pdeB::Km, since the c-di-GMP-degrading PDE enzyme has been knocked out. Complementation with the WT gene led to a drastic 11-fold decrease in intracellular c-di-GMP levels for C-pdeA and a 2.4-fold decrease for C-pdeB. In both the akrA::Km and C-akrA strains, the intracellular c-di-GMP content was similar to that of the WT strain.

### *Leptospira*’s biofilm does not promote virulence but protects against environmental stresses

**Fig. 5:**
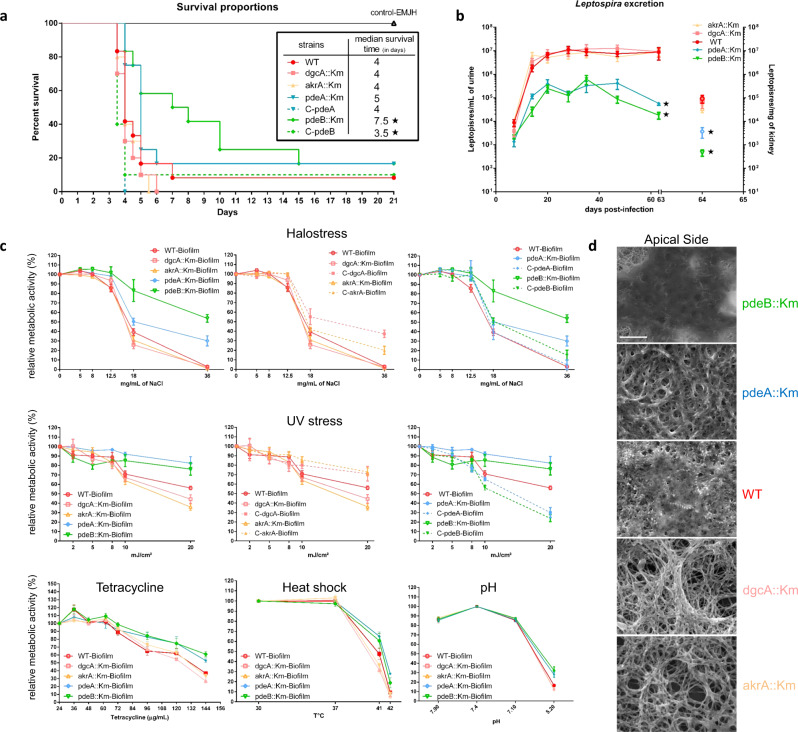
*Leptospira* biofilm protects bacteria against environmental-like conditions. Solid lines represent mutant strains and dotted line complemented strains. Mutant akrA::Km is shown in orange, dgcA::Km in pink, pdeA::Km in blue and pdeB::Km in green. Control EMJH-injected hamsters are shown in black. Error bars represent the standard error of the mean. **a** Survival rate over 21 days for hamsters challenged with c-di-GMP mutants and WT strain. * Indicates a *p* value < 0.05 in Gehan–Breslow–Wilcoxon test. The table recapitulates the median survival time for each strain. **b** Quantification of *Leptospira* excretion over 63 days for mice challenged with c-di-GMP mutants and WT strain expressed as the number of *Leptospira* per mL of urine after challenge (plotted on the left axis). *Leptospira* kidney load in mice challenged with c-di-GMP mutants and WT strain is expressed as the number of *Leptospira* per mg of kidney at day 64 post-inoculation (plotted on the Right axis). **c** Relative metabolic activities of WT and c-di-GMP mutant biofilms under halostress, UV stress, tetracyclin treatment, heat shock and exposure to various pH. Relative metabolic activities are expressed as a percentage of the metabolic activity of the untreated WT strain (Halostress, UV stress, Tetracyclin) or WT strain under standard culture conditions (Heat and pH). Untreated, 3 weeks old biofilms were used as a positive control for metabolic activities (100%). The effect of simulated environmental stresses on treated biofilm were expressed in percentage of the control condition. Mean of at least five independent experiments are shown; error bars represent standard errors of the mean. **d** SEM micrograph illustrating matrix production by the different c-di-GMP mutants as compared to the WT strain. * Indicates a *p* value < 0.05 in two-tailed unpaired Mann–Whitney test compared to the WT strain. Scale bar: 5 µm.

We next looked at whether *Leptospira*’s ability to produce a biofilm was involved in virulence or colonization, using a hamster model of acute disease and a mouse model of chronic renal carriage. In hamsters infected with pdeB::Km (i.e. strain with high biofilm production but low motility), the median time to death (7.5 days) was longer than in WT infections (4 days) (Fig. 5a). The median time to death was also 4 days for the dgcA::Km and akrA::Km strains. Complementation of pdeB::Km with the WT gene substantially restored virulence, with a median time to death of 3.5 days.

Chronic carriage in mice was investigated by quantifying the bacterial load in urine and kidney (Fig. 5b). The bacterial loads in both urine and kidney were lower for pdeA::Km and pdeB::Km than for the WT—reflecting a lower colonization capacity. In contrast, the bacterial loads for dgcA::Km and akrA::Km were similar to those observed for the WT.

We investigated the biofilm’s role as a protective barrier against external stresses by exposing mature biofilms to simulated environmental stresses: elevated NaCl concentrations, ultraviolet light, the antibiotic tetracycline, heat, and acidic/basic pH conditions (Fig. 5c). The degree of protection was assessed by measuring the metabolic activity of the bacteria contained in the biofilm after stress exposure. Our results revealed that regardless of the type of stress, the two biofilm-over-producing mutants (pdeA::Km and pdeB::Km) always displayed a higher level of cellular activity than biofilm-under-producing mutants (dgcA::Km and akrA::Km) (Fig. 5c); the WT strain was positioned between these two groups. Furthermore, we observed that biofilm-forming bacteria were less sensitive to stress than their planktonic counterparts (Supplementary Fig. 3A). In particular, we found that biofilm-over-producing mutants survived exposure to the concentration of salt (36 mg/mL) found in sea water. Following exposure to intense UV light (20 mJ/cm²), the metabolic activity of biofilm-forming bacteria remained exceptionally high. In line with the results described above, complementation with the WT gene reverted the mutants’ ability to withstand NaCl and UV exposure (Fig. 5c). Each strain’s metabolic activity after NaCl or UV exposure was positively correlated with its biofilm production capacity (Supplementary Fig. 3B). Lastly, we examined the biofilm produced by these four mutants at the cellular level. Scanning electron micrographs (Fig. 5d) highlighted significant interstrain differences in ECM production. The pdeA::Km and pdeB::Km mutants were deeply enmeshed within a thick matrix that prevented individual bacteria from being seen, whereas individual bacteria could easily be seen in the ECM-poor biofilm produced by the dgcA::Km and akrA::Km mutants.

## Discussion

Our multidisciplinary analysis of the initial steps in biofilm formation provided detailed information on the strategies used by pathogenic *L. interrogans* to create resilient biofilms and thus protect itself against extracellular stresses. We also showed that the c-di-GMP pathway is functional in *L. interrogans* and has a pivotal role in motility and biofilm formation in this bacterium.

Firstly, we described the sequence of events that leads to the formation of a mature biofilm (Fig. 1). Furthermore, we demonstrated conclusively that the *L. interrogans* biofilms primarily contain live bacteria (Fig. 1c), and are not haphazard aggregates of sedimenting dead cells or cell debris.

We combined live and high-resolution imaging techniques to reveal differences in biofilm formation in *Leptospira*, relative to well-characterized structures produced by other bacteria^15^ (Fig. 1d, e, g). For example, we found that the initial steps in *Leptospira* biofilm formation result from the accretion of bacterial aggregates. Formation of these bacterial aggregates is not fully elucidated and the relative contribution of clonal growth and/or recruitment of planktonic cells in biofilm growth would deserve further studies.

The initial aggregates did not adhere firmly to the substratum and were exceptionally mobile for multicellular microbial communities. Similar observations have been made with flocs that settled on the substratum and kept moving solely based on connective currents or Brownian motion. However, leptospires are known to be fast swimmers and also able to crawl on a solid surface^34^. This particular crawling motility could explain the movement of these multicellular aggregates and might challenge the current paradigm of a sessile biofilm attached to a surface.

We observed a number of unique organizational features. The *Leptospira* in the aggregates were able to organize with each other (Supplementary Fig. 1C and Supplementary Movie 2), which is suggestive of an efficient bacterial communication system. This particular cell migration produced a mushroom-like structure with an empty centre (Fig. 1g). Imaging of both the basal and apical sides of the mature biofilm (Fig. 2c, e) revealed a highly polarized, structurally organized biofilm. The latter’s architectural features (such as interstitial voids and channels, resulting in a foam-like structure) appears well suited to the diffusion of nutrients, gases, and other metabolites^35^ required for bacterial life (Fig. 2f, g). As seen in our SEM experiments, the *Leptospira* biofilm was massive (>1 cm long and several hundred µm thick) after 3 weeks of culture. The interconnected channels can be likened to a microcirculatory system, and might favour further growth of the biofilm community by ensuring the influx of nutrients and oxygen and the efflux of waste products^36^. On the exposed side of the biofilm (i.e. at the interface with the environment), the leptospires were covered by a thick matrix, rather than the fibrous casing^37^ or curli fibers^38^ observed in other species. The presence of budding-like structures at the surface of the biofilm might be suggestive of a dissemination mechanism via spread of the biofilm progenitor’s structures (Fig. 2h).

Our CLSM results showed that exDNA is one of the main ECM constituents of *Leptospira*’s biofilm matrix (Fig. 3a). Furthermore, the effect of DNase I treatment highlighted the exDNA’s key role in maintaining a dense biofilm (Fig. 3b). In several pathogenic bacteria (such as *Listeria monocytogenes*, *Enterococcus faecalis*, and *Pseudomonas aeruginosa*), exDNA is a major biofilm component^39^ and autolysis is considered to be one of the sources of exDNA^40^. Our results also showed that the *L. interrogans* biofilm matrix also contains extracellular polysaccharides (EPSs), although we were not able to determine the type (Supplementary File 6). Although alginate is hypothetically a constituent of the *Leptospira* biofilm^41–43^, our use of alginate lyase did not trigger biofilm dissolution—indicating that alginate is not the main EPS constituent.

Recent work^30^ suggested that the c-di-GMP regulatory pathway is functional in *L. interrogans*. Furthermore, the c-di-GMP pathway is known to be regulated during biofilm formation by *Leptospira biflexa*^43^.

By studying *Leptospira* mutants with defects in c-di-GMP synthesis or degradation, we demonstrated that impaired c-di-GMP synthesis resulted in a low intracellular concentration of this metabolite (Fig. 4g). In turn, this resulted in low biofilm production (Fig. 4a) and enhanced motility (Fig. 4f). Although motility is a virulence factor in *L. interrogans*, we could not demonstrate a greater virulence in the animal models studied here (Fig. 5a). However, defects in biofilm production resulted in a lower degree of protection against simulated environmental stresses (Fig. 5c). This sensitivity was probably due to the massive relative reduction in the quantity of ECM –thought to act as a shield (Fig. 5d). Conversely, low phosphodiesterase enzymatic activity led to the intracellular accumulation of c-di-GMP (Fig. 4g), massive biofilm production (Fig. 4c, d), and low motility (Fig. 4f). In a hamster model, pdeB::Km showed a low level of virulence—possibly also a consequence of the loss of motility (Fig. 5a). We had expected greater biofilm production to lead to more effective colonization of the kidneys. However, chronic carriage in mice was low for the pdeA::Km and pdeB::Km mutants. The low observed urine levels did not result from greater retention of leptospires in the kidneys because the latter were less colonized than in a WT infection (Fig. 5b). This finding may be partly explained by the low levels of motility and virulence, which probably impaired the bacterium’s ability to infect the host, spread in the body, and colonize the renal tubules^44^. Nevertheless, both PDE mutants were extremely resistant to environmental stress (Fig. 5c), which was correlated with a thick biofilm ECM (Fig. 5d).

In conclusion, we used transposon mutants to demonstrate that DGC/PDE activities control intracellular c-di-GMP levels, which regulate motility and biofilm production in *Leptospira interrogans*. We showed that the intracellular accumulation of c-di-GMP is associated with enhanced biofilm production and greater ability to withstand environmental stresses. Once a mature biofilm has developed, it can be extremely difficult to eradicate *Leptospira*. The biofilm is a highly polarized, organized structure that successfully provides life-compatible conditions—notably due to the protective ECM layer and a complex network of voids for nutrient and gas exchanges. This is particularly true for saprophytic species, whose biofilm production is correlated with the ability to persist in the environment. Indeed, in vitro biofilm production is mostly prominent for *Leptospira* species in the saprophyte subclade S1 (Supplementary Fig. 3C). This is very interesting from an ecological viewpoint, since these species are fully adapted to an exclusively environmental lifestyle. In addition, environmental conditions such as low temperature, low glucose and nutrients, and oxidative stress are known to maximally induce biofilm formation genes as compared to host-associated conditions^45^. Of note, the optimum temperature of *Leptospira* is 30 °C rather than 37 °C like most pathogenic bacteria. The fact that multimicrobial environmental biofilms can spontaneously harbour *Leptospira* (including pathogenic species) in situ^9^ may explain why viable, virulent *Leptospira* can be found after prolonged periods of time in the environment^4^.

Lastly, by regulating biofilm formation, c-di-GMP signalling can also influence *Leptospira’*s ability to resist a wide variety of stresses. Manipulating and subverting c-di-GMP signalling might compromise biofilm formation, antimicrobial tolerance, and *Leptospira*’s persistence—thus opening up perspectives for the control of leptospirosis^46,47^.

## Methods

### Bacterial strains, culture conditions and transposon mutagenesis

*Leptospira* strains used in this study, including *Leptospira interrogans* serogroup Pyrogenes serovar Manilae strain L495 designated in this study as the wild type (WT) or parental strain, are listed in Supplementary Table 3 and were cultivated in Ellinghausen and McCullough as modified by Johnson and Harris (EMJH^48,49^) growth medium at 30 °C under static conditions. For virulent strains, cultures were regularly passaged into hamsters and used until a maximum of five in vitro passages. Mutants dgcA::Km (LMANV2_v2_150005 || Frame -2 || Begin: 1223263 || End: 1224255 || Length:993), akrA::Km (LMANV2_v2_50029 || Frame +3 || Begin: 242451 || End: 243287 || Length:837), pdeA::Km (LMANV2_v2_270021 || Frame +1 || Begin: 2272684 || End: 2274402 || Length:1719) and pdeB::Km (LMANV2_v2_90001 || Frame -2 || Begin: 631792 || End: 633144 || Length: 1353) were generated from the parental strain *L. interrogans* serovar Manilae strain L495 by transposon mutagenesis as described previously^50,51^ (Supplementary Fig. 4A) and grown under the same conditions. Transposon insertion was confirmed by PCR using primers flanking the insertion sites (Supplementary Fig. 4B). Complementation was performed as described previously by cloning genes into the spectinomycin resistant pMaORI plasmid to reintroduce the functional gene under the control of its native promoter^52^. Similarly, an empty pMaORI plasmid was also introduced into the mutants as a control (Supplementary Table 3). Complemented mutants were cultivated under selection pressure (50 μg/mL of Spectinomycin).

### Biofilm formation

Bacterial strains were grown to exponential phase and counted using a Petroff-Hausser cell-counting chamber (Hausser Scientific Company, Horsham, PA, USA) under dark-field microscopy. Cultures were diluted in EMJH to a final concentration of 1 × 10^6^ bacteria mL^−1^. Under sterile conditions, 150 μL of diluted cultures were dispensed into the wells of a 96-well microtiter polystyrene plate (flat bottom, with low evaporation lid, Nest Biotechnology). As border effect is known to occur, the peripheral wells of the plate were not used, but instead filled with sterile water to minimize evaporation. For quantitative assays, 18 replicate wells were used for each strain. Microtiter plates were then incubated at 30 °C for 3 weeks under static conditions without changing the culture medium.

### Crystal Violet (CV) staining and quantification

After 3 weeks of static incubation, planktonic cells were removed by gently pipetting the supernatant. The biofilm was gently rinsed with 200 μL of PBS (137 mM NaCl, 2.7 mM KCl, 8 mM Na_2_HPO_4_, and 2 mM KH_2_PO_4_) at room temperature. Fixation was performed by incubation with 150 μL of 4% paraformaldehyde (PFA) in PBS at room temperature for 30 min. Fixative buffer was then removed and biofilm was rinsed two times with 200 μL of PBS prior to staining with 175 μL of 0.1% CV solution. After 10 min of incubation, the CV solution was removed. Biofilms were rinsed two times with 200 μL PBS and dried overnight. The stain was then released with 200 μL of destaining solution [50% (vol/vol) ethanol, 50% (vol/vol) glacial acetic acid]. The amount of stain released was quantified by measuring the absorbance at 570 nm with a microplate reader (Multiskan FC, Thermos Scientific). Alternatively, phase contrast images of stained biofilms were captured using an upright Leica DM4000 B microscope (Leica microsystems, Mannheim, Germany).

### Quantification of biofilm formation by image analysis

Biofilm quantification was performed from phase contrast images using the ImageJ software^53^ (https://fiji.sc/). Phase contrast images of the growing biofilm were acquired using an upright Leica DM4000 B microscope equipped with a 5X lens (HCX PL FLUOTAR 5×/0,15, 12 mm working distance). Pictures of six replicate wells per independent experiment were acquired and further analysed. Prior to quantification, the outer part of the image affected by spherical aberrations and field curvature artefacts was cropped. The 2D grayscale image was then binarized and analysed using the “Analyse Particles” function. The biofilm score was then expressed as a percentage of surface occupied by the biofilm. Alternatively, we quantified biofilm growth by CV solubilisation and absorbance measurement at 600 nm using a spectrophotometer (Multiskan FC, Thermos Scientific). Both methods captured correctly the increase of biofilm formation over time (Supplementary Fig. 5). In this study surface-based method was chosen for its better measurement accuracy and the advantage of limiting sample handling, thereby reducing erroneous quantification.

### Time-lapse imaging

Time-lapse imaging of growing biofilm was acquired using the BioStation IM-Q Inverted Microscope (Nikon, Tokyo, Japan). Five hundred μL of a bacterial suspension at 10^6^ bacteria mL^−1^ were inoculated into a 35-mm glass bottom Hi-Q4 dish (Ibidi, Martinsried, Germany). Glass bottom Hi-Q4 dish incorporate a plane parallel top plate, preventing light path distortion by the meniscus, therefore enabling high-quality phase contrast observations. Time-lapse imaging started 12 h after seeding under static condition at 30 °C and 95% of humidity. Phase contrast images were acquired every 30 min over a period of 9 days using a 20X objective (field of view: 413*311 µm).

### Confocal laser scanning microscopy

Biofilms were prepared as described in the Biofilm Formation section except inoculation performed in µ-dish 35 mm (Ibidi, Martinsried, Germany). Biofilms were fixed in 4% PFA/PBS solution for 30 min or stained using the LIVE⁄DEAD Biofilm Viability Kit (Invitrogen™), wheat-germ agglutinin (WGA, 10 μg/mL) conjugated to Fluorescein (Invitrogen™), Concanavalin A (Con A, 10 μg/mL) conjugated to Fluorescein (Invitrogen™), BOBO-3 (3 nM, Invitrogen™), or the FilmTracer SYPRO Ruby Biofilm Matrix Stain (Invitrogen™), as described by the manufacturer’s instructions. Staining with the Vybrant™ CFDA/SE Green Cell Tracer (10 μM, ThermoFisher Scientific) was performed prior to fixation. According to the manufacturer, WGA binds N-acetyl-d-glucosamine and N-acetylneuraminic acid residues, Con A selectively binds to x-mannopyranosyl and x-glucopyranosyl residues, BOBO-3 is a cell-impermeable DNA stain, and SYPRO Ruby stain labels most classes of proteins. The stained biofilms were visualized using an inverted SP8 confocal laser-scanning microscope (Leica Microsystems, Mannheim, Germany). A super continuum white light laser tuneable between 470 and 670 nm was used for excitation and focused through a HC PL APO CS2 63×(N.A 1.4) oil immersion lens. Emission signals were captured by hybrid detectors. The system was controlled by Leica Application Suite X (LAS) software. 3D images were directly processed using LAS X, 3D module software.

### Scanning electron microscopy

*Leptospira* strains were cultured as described in the biofilm formation section with minor modifications. Inoculation was performed by adding one mL of diluted culture in a 24-well plate (flat bottom, with low evaporation lid, Corning) each well containing a sterile 12 mm Glass coverslips (12 × 12 mm, Menzel-Glaser, Braunschweig, Germany). The supernatant was removed and biofilm was rinsed once in PBS, then fixed in 4% PFA / 1% glutaraldehyde for 15 min. Coverslips were rinsed again in PBS and stained with 1% osmium tetroxide (OsO_4_ in PBS, Acros Organics) for 1 h. After staining, samples were dehydrated through a series of ethanol concentrations (25, 50, 70, 90, and 100% for 10 min each) before being desiccated using hexamethyldisilazane (HMDS, Acros Organics). HMDS drying is a reliable alternative to critical point drying in the preparation of the samples with no differences in term of distortion and shrinkage between the two methods regarding cellular ultrastructure^54^. Before visualization, samples were submitted to carbon sputtering to increase their electrical conductance (Em ACE600, Carbon Coater, Leica Microsystems, Mannheim, Germany) and examined under a Jeol JSM IT-300 IntouchScope microscope (SED detector, 10 kV, Jeol LTD, Tokyo, Japan).

### Dissociation of biofilms by chemical or enzymatic treatments

WT biofilms were grown as described in the Biofilm Formation section, and after 21 days of incubation, twenty µL of DNase I (6.25–500 µg/mL in 150 mM NaCl, 1 mM CaCl_2_; 10104159001; Roche Diagnostics GmbH, Manheim, Germany), 20 µL of proteinase K (6.25–500 µg/mL in 50 mM Tris-HCl pH 7.5, 1 mM CaCl_2_; 05323738001; Roche Diagnostics GmbH, Manheim, Germany), 20 µL of alginate lyase (1–200 U in 20 mM Tris, 200 mM NaCl; A-1603-100MG, Sigma Aldrich, St-Louis, MO, USA) and 20 µL of sodium periodate (1–100 mM in 50 mM sodium acetate; S1878-25G, Sigma Aldrich, St-Louis, MO, USA) were added directly to the biofilms. Control wells were treated with 20 µL of the corresponding buffer only. Wells were incubated at 30 °C for 48 h. Phase contrast images of adherent biofilms were captured with an upright Leica DM4000 B microscope (5X objective HCX PL FLUOTAR 5×/0,15,) using a DFC450 digital CCD camera (Leica Microsystems, Mannheim, Germany).

### C-di-GMP extraction and quantification

Forty mL of a 10^6^ bacteria mL^−1^ suspension were inoculated into a 50 mL culture flask (Falcon) during 21 days at 30 °C under static conditions. Twenty mL were used for c-di-GMP extraction and 20 mL were used for normalization. Bacterial suspensions were centrifuged at 2500 × *g* for 20 min at 4 °C. For c-di-GMP extraction, bacterial pellets were re-suspended in 300 μL of ice-cold extraction solution (acetonitrile/methanol/water; 2/2/1; v/v/v) for 15 min on ice prior to heat extraction at 95 °C during 10 min. After cooling, the suspension was centrifuged at 20,000 × *g* for 10 min at 4 °C in order to separate insoluble material from the extracted nucleotides. The extraction procedure was repeated twice. The supernatant was collected and then evaporated until dryness at 30 °C using a Speed Vac (Concentrator plus, Eppendorf). The pellet was re-suspended in 200 μL of water using vigorous vortexing and then analysed by liquid chromatography (Series 200, Perkin Elmer, Norwalk, CT, USA) coupled with an API 3000 triple quadrupole mass spectrometer (Concord, Ontario, Canada) as described elsewhere^55^.

### Growth rate measurements

Mutants, complemented and WT strains were counted using a Petroff-Hausser counting chamber under dark-field microscopy. Five mL of EMJH inoculated at 1.10^6^ bacteria mL^−1^ of each strain were cultured over a 9-day period under static conditions. Growth was monitored at 30 °C by counting bacteria every day using a Petroff-Hausser counting chamber. Growth experiments were performed three times on different days and using independent cultures.

### Bacterial speed displacement quantification

Bacterial strains were grown to exponential phase and 10 μL of bacterial suspension adjusted at 10^6^ bacteria mL^−1^ were prepared for dark-field contrast microscopy using a #1.5H square coverslip. Motility was assessed in liquid medium by video microscopy using a BX53 Olympus microscope (Olympus Corporation, Nishi-Shinjuku, Tokyo, Japan) equipped with a Hamamatsu 2.8 Orca flash camera (Hamamatsu Photonics, Hamamatsu City, Japan). For each strain, three experiments were performed on different days and using independent cultures. In total, 190 bacteria per strain were recorded over 60 s. Trajectory analysis and speed displacement were calculated using Olympus CellSens Dimension imaging software.

### Animal infection and quantification of bacterial burden

1.10^8^ leptospires in EMJH were inoculated intraperitoneally into 7–8-week-old golden Syrian hamsters (males and females) and 8-week-old Oncins-France 1 (OF-1, outbred) mice (males and females) whose progenitors originated from Charles River Laboratories. Hamsters were monitored twice daily for 21 days and subsequently euthanized by carbon dioxide inhalation. During follow-up, hamsters displaying poor or no reaction upon stimulation were humanely euthanized and considered dead. Kidneys were collected from animals sacrificed at day 21. Mice were monitored during 63 days and urine was collected at days 7, 14, 21, 28, 35, 47, 63 after infection. At day 64 mice were euthanized by carbon dioxide inhalation and kidneys were collected. DNA was extracted from urine (50 μL) and kidney (25 mg) and analysed by a quantitative PCR targeting the conserved regions of the 16S rRNA gene^56^. A PCR targeting the pMaORI plasmid was performed at the end of the 63 days of infection to ensure that complemented mutants were still carrying the complementation plasmid (primers pMaORI-1F, 5′-TCG-ATT-GGT-GTA-GTC-GGT-T-3′; pMaORI-1R, 5′-GCA-GCC-ATT-CAA-TTT-CTT-GAG-TTA-3′). Four experiments (12 animals) per condition were performed on different days and using independent bacterial cultures. Animal experiments were conducted according to the guidelines laid out by the Animal Care and Use Committee of the Institut Pasteur of Paris and of New Caledonia, and European Recommendation 2007/526/EC. Protocols and experiments were approved by the Animal Care and Use Committee of the Institut Pasteur of New Caledonia.

### Susceptibility assay

*Leptospira* strains were cultured as described in the biofilm formation section. For each strain tested, six out of eighteen wells were vigorously resuspended by pipetting to mechanically disrupt the biofilm, in order to put the bacteria back in suspension. These wells were used to compare biofilm with planktonic cells with a similar metabolic status after three weeks of culture. Environmental-like stress was applied: NaCl (0, 5, 8, 12.5, 18 and 36 mg/mL); UV light (0, 2, 5, 8, 10, 20 mJ/cm²); tetracycline (24, 36, 48, 60, 72, 84, 96, 108, 120, 132, 144, 156 μg/mL); temperature (30, 37, 41, 42 °C) and pH (5.2, 7.1, 7.4, 7.9). After 24 h of stress exposure, 30 μL of resazurin solution (alamarBlue Cell Viability Reagent, Life Technologies SAS) was added to each well, and the plates were incubated for 48 h at 30 °C. The chromogenic shift was measured by absorbance at 570 nm/620 nm with a microplate reader (Multiskan FC, Thermos Scientific). Negative controls without bacteria were included. Relative metabolic activity was calculated as recommended by the manufacturer and expressed in percentage. Experiments were repeated at least 4 times.

### Statistical analysis

All statistical analyses were performed with GraphPad Prism version 7.0 (GraphPad Software, San Diego, CA). The statistical tests used and *P* values are indicated within the text and figure legends. All experiments were performed a minimum of three times on separate days in duplicate or more.

### Reporting summary

Further information on experimental design is available in the Nature Research Reporting Summary linked to this article.

## Supplementary information

Supplementary Movie 1

Supplementary Information

Supplementary Movie 2

Reporting Summary

## Data Availability

The data that support the findings of this study are available from the corresponding author upon reasonable request.
