# Supplementary material for: The zoonotic pathogen Leptospira interrogans mitigates environmental stress through cyclic-di-GMP-controlled biofilm production
Source: NPJ Biofilms Microbiomes. 2020 Jun 12;6:24. doi: 10.1038/s41522-020-0134-1 (PMC7293261; doi:10.1038/s41522-020-0134-1)
Supplement: Supplementary file 2 — Supplementary Information [file 41522_2020_134_MOESM2_ESM.pdf]

Supplementary Fig 1

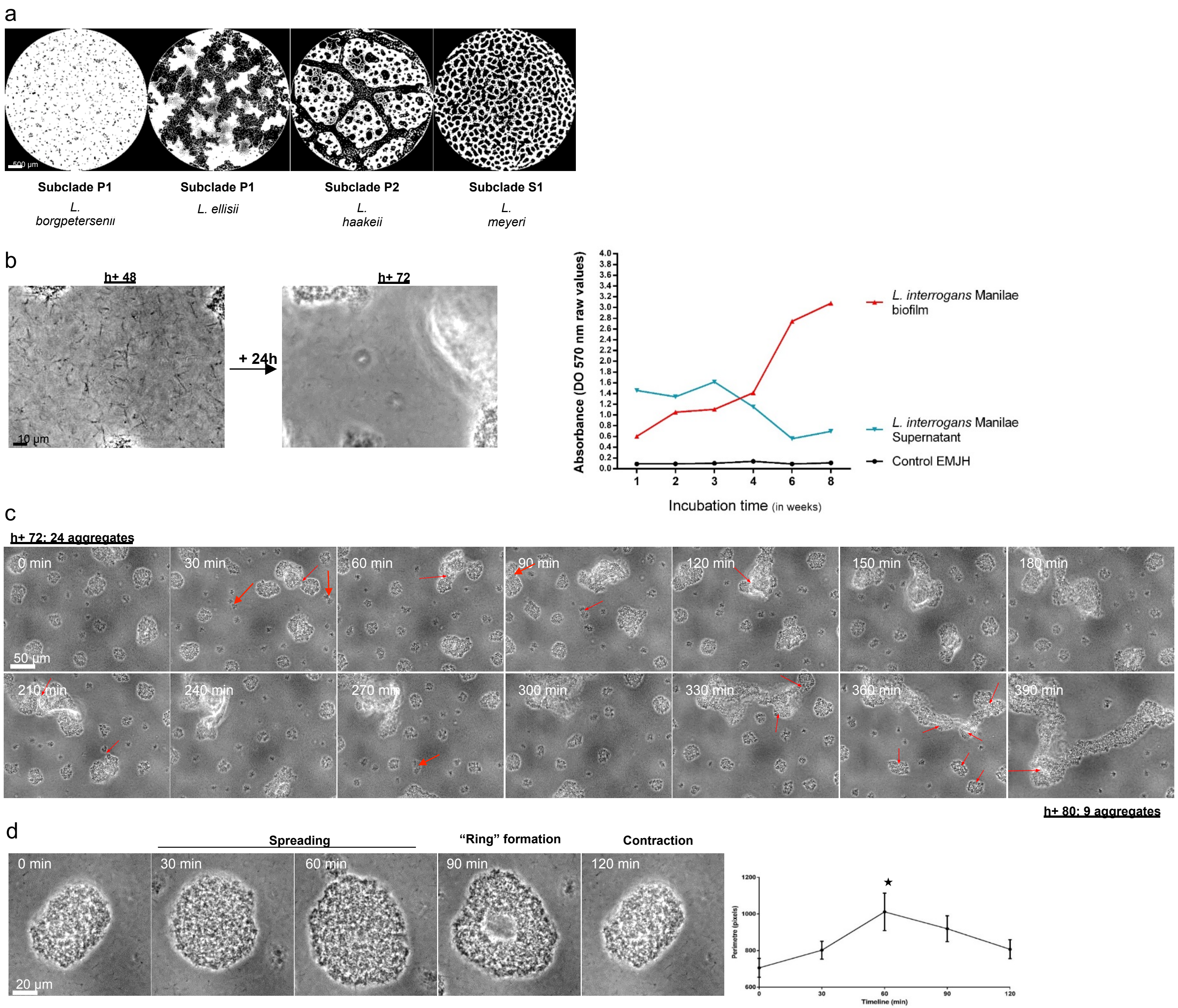

Legend Supplementary Fig 1

Supplementary Figure 1.
(a) Phase contrast images of the biofilm of Pathogenic subclade P1 (*L. borgpetersenii*, *L. ellisii*), Pathogen subclade P2 (*L. haakeii*) and Saprophyte subclade S1 (*L. meyeri*) *Leptospira* at 3 weeks post-inoculation. (b) Phase contrast images of *Leptospira* planktonic bacteria at 48 and 72h (left panel) and the corresponding quantification of the amount of planktonic bacteria and bacteria associated to the biofilm. Raw absorbance values of Crystal Violet (490 nm) staining associated to the supernatant (planktonic bacteria) and to the substrate (biofilm-associated bacteria) is plotted for *L. interrogans* Manilae and the negative control EMJH (black, right panel). (c) Sequence of phase contrast images of biofilm coalescence between 72 and 80h post-inoculation. (d) Sequence of phase contrast images of the synchronized spreading and contraction behaviour delineating cavities within biofilm aggregate (left panels) and quantification of the average perimeter of biofilm aggregates. * indicates a p value < 0.05 in two-tailed unpaired Mann-Whitney test.

Supplementary Fig 2

A.

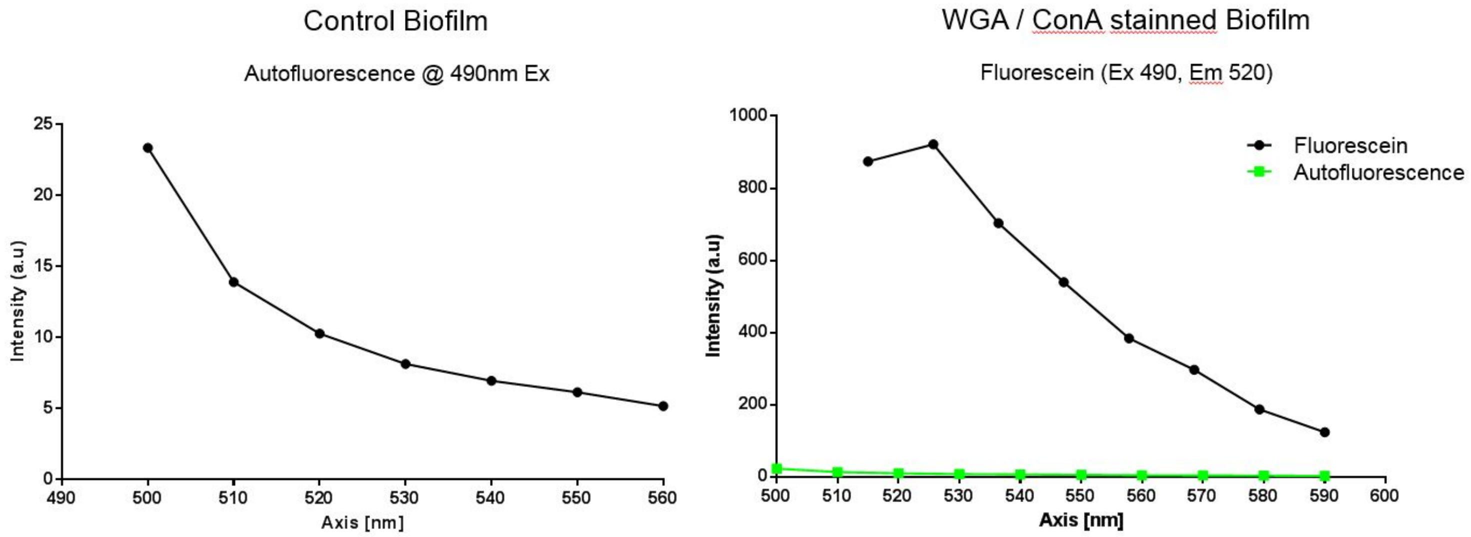

B.

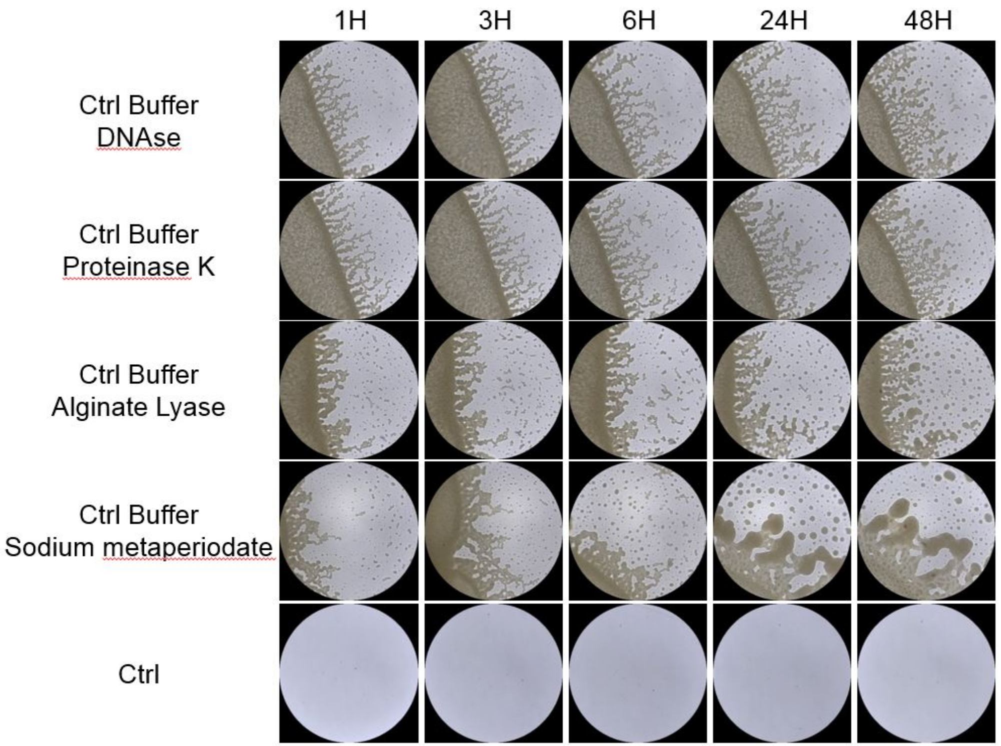

Legend Supplementary Fig 2

Supplementary Figure 2.

(a) Spectral evolution of the biofilm light emission after excitation at 490 nm. Left panel show the light emission of the unstained control biofilm while the right panel reveal the light emission stained with the WGA/ConA-fluorescein. Note the 9-fold increase at the fluorescein maximum emission peak (520 nm). (b) Phase contrast images of biofilms incubated for 1, 3, 6, 24 and 48h with the activation buffer used for the dissociation assay.

Supplementary Fig. 3

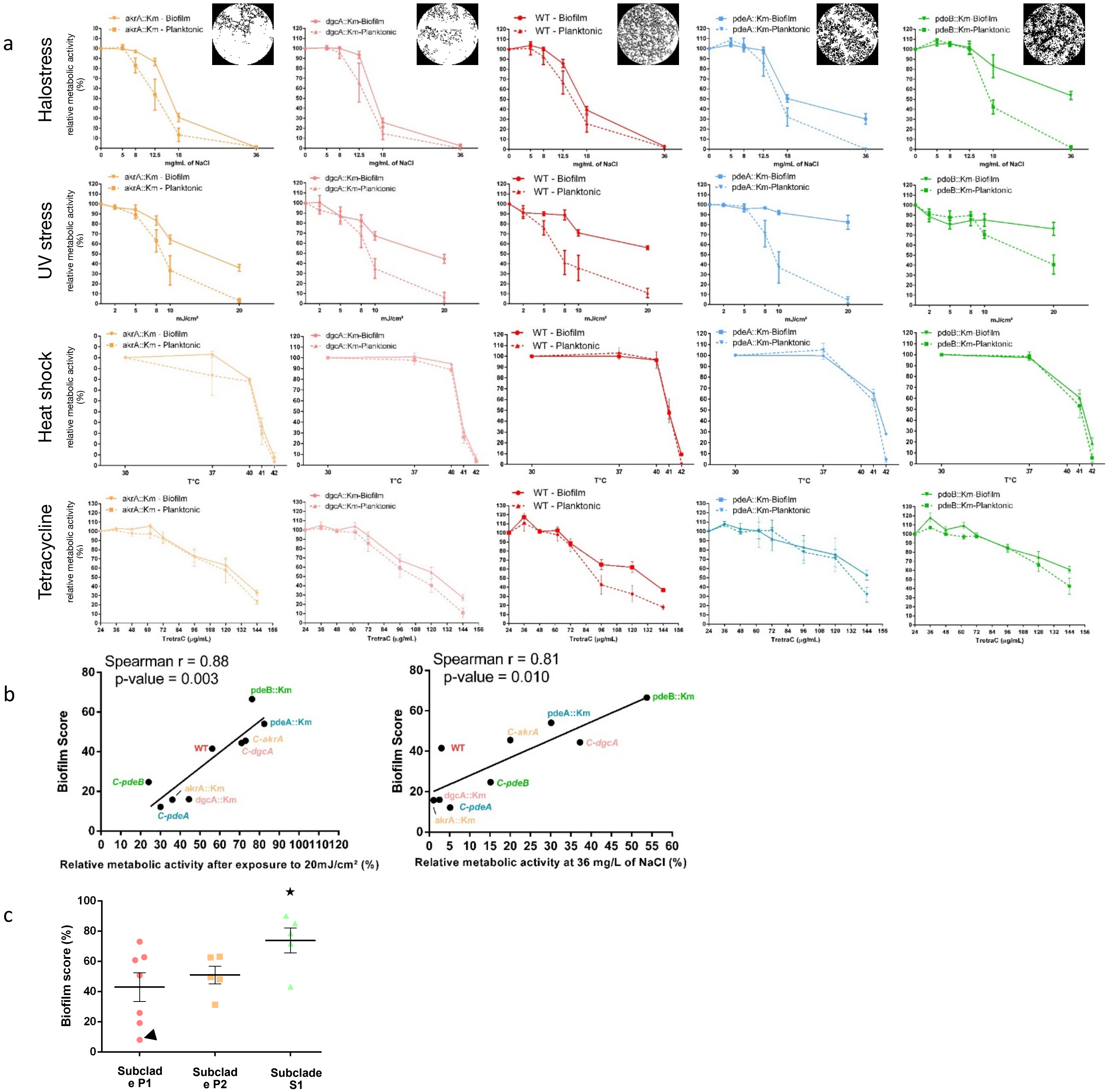

Supplementary Figure 3.

(a). Relative metabolic activities of WT and C-di-GMP mutant planktonic bacteria under halostress, UV stress, tetracycline treatment and heat shock as compared to their biofilm-associated counterparts. Relative metabolic activities are expressed as a percentage of the metabolic activity of the untreated WT strain (Halostress, UV stress, Tetracycline) or WT strain under standard culture conditions (Heat). Mean of at least five independent experiments are shown; error bars represent standard errors of the mean. (b) Correlation analysis between Biofilm score expressed as the percentage of well surface colonized by biofilm and relative metabolic activities after exposure to 36 mg/ml of NaCl (left panel) and 20 mJ/cm^2 (right panel). (c) Biofilm score expressed as the percentage of well surface colonized by biofilm for subclades P1, P2 and S1. Each dot represents a different *Leptospira* species. Note that *L. borgpetersenii* (arrowhead) is the species that produced the least biofilm under our experimental design. This species belongs to the pathogenic subclade P1, and has a reduced genome (lacking several genes involved in exopolysaccharide production) as a result of full adaptation to its mammal host and evolution towards a parasitic lifestyle - resulting in poor ability to survive in the environment. Means and standard errors of the means are plotted. * indicates a p value < 0.05 in two-tailed unpaired Mann-Whitney test.

Supplementary Fig. 4

a.

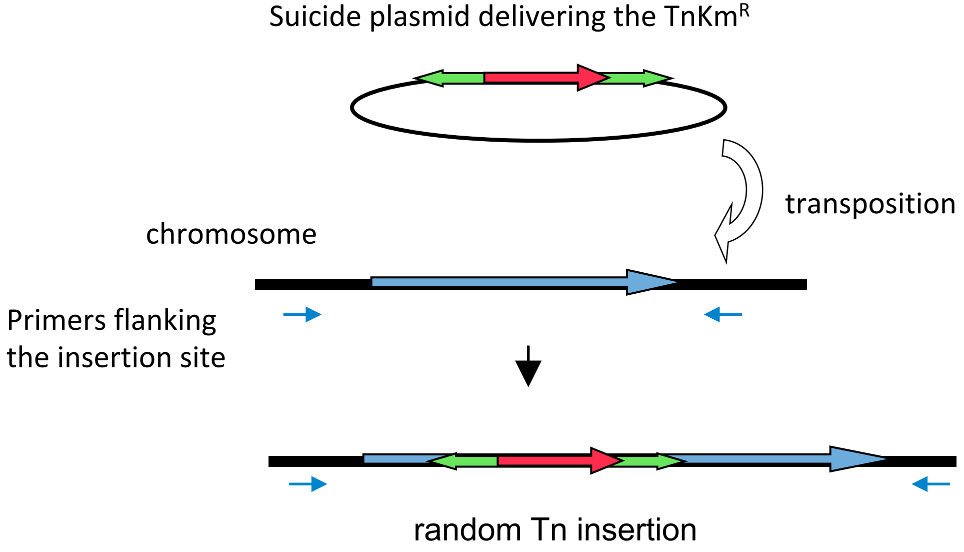

b.

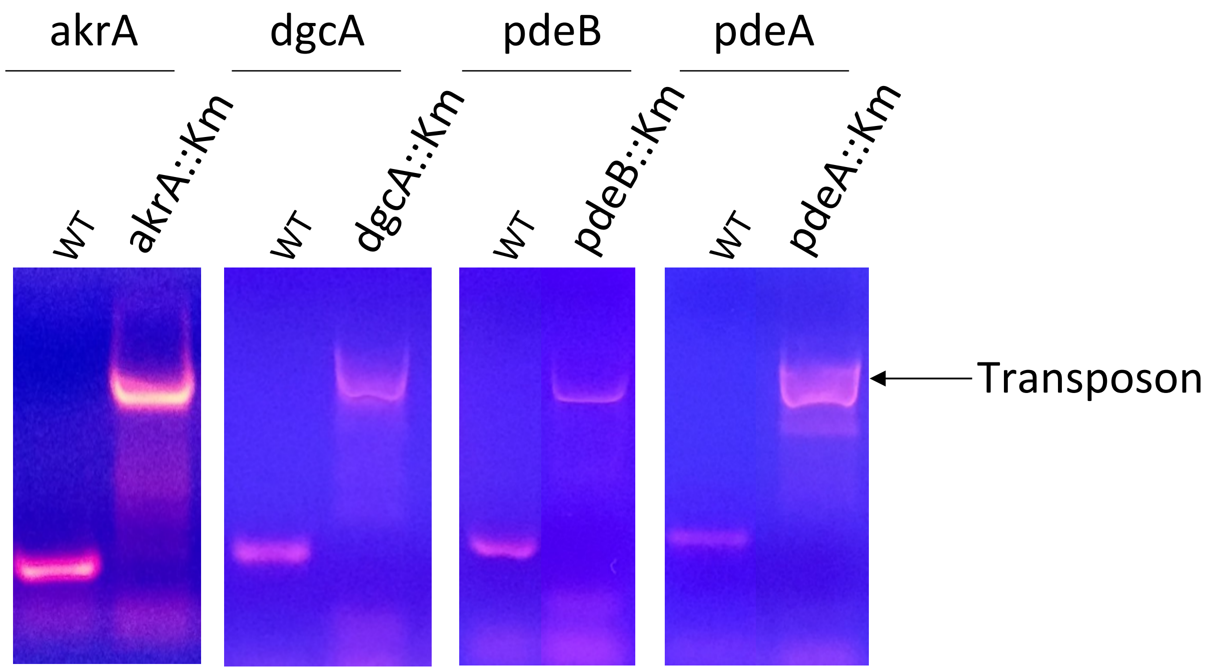

Legend Supplementary Fig 4

Supplementary Figure 4. Transposon mutagenesis strategy and confirmation.
(a) Simplified diagram illustrating the mutagenesis strategy based on transposon insertion. Primers flanking the insertion site were used to confirm transposon insertion within the gene of interest. (b) Transposon insertion was analysed by PCR and amplified product were subjected to electrophoresis in a 2.0% agarose gel to reveal the presence of the inserted transposon. Gels derive from the same experiment and were processed in parallel.

Supplementary Fig. 5

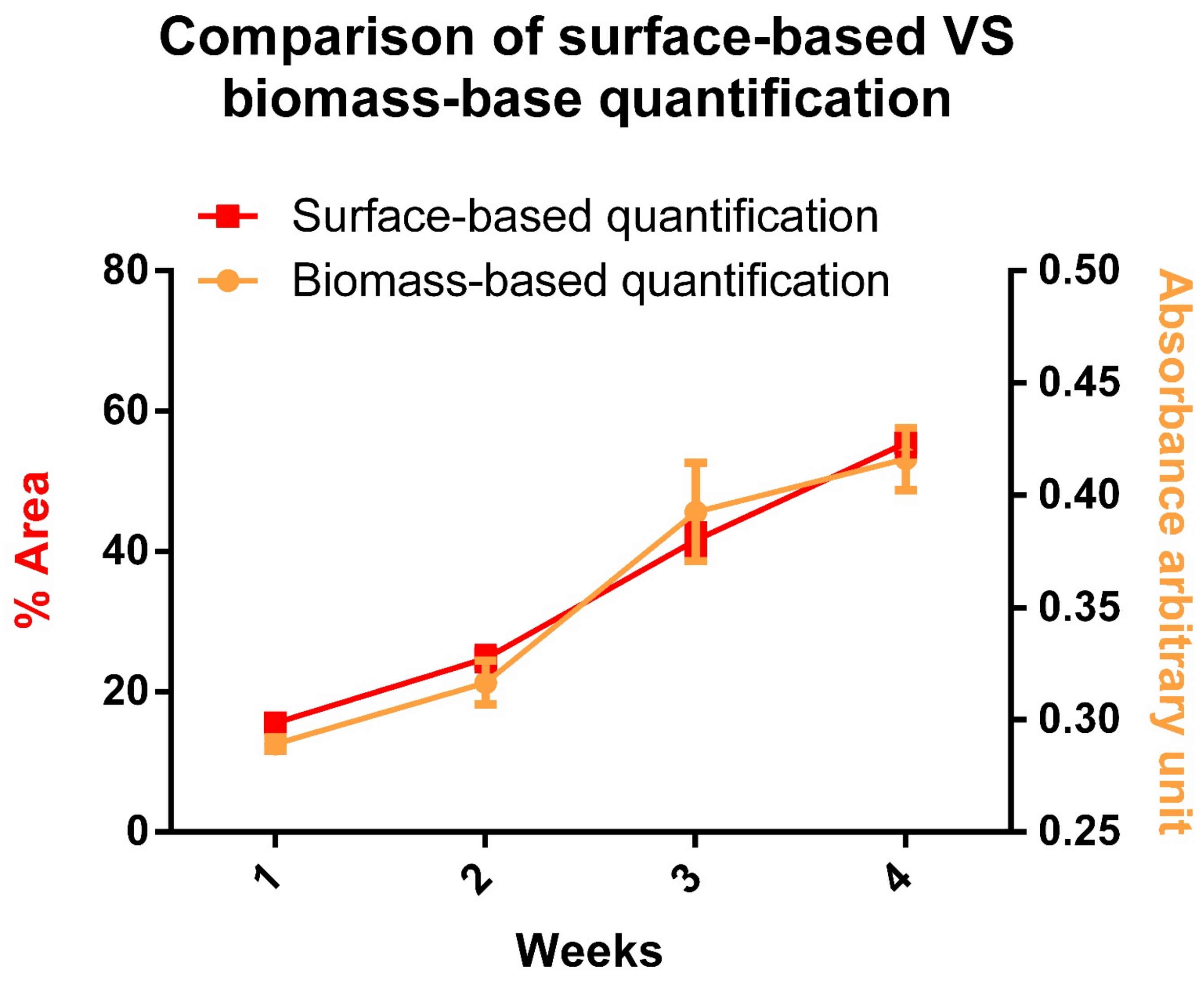

Legend Supplementary Fig. 5

Supplementary Figure 5. Biofilm quantification over time.

The graphic illustrates the two methods used to quantify *Leptospira* biofilm production over time. The surface-based method (red, left Y-axis) relying on phase contrast image analysis and the biomass-based method (Orange, right Y-axis) relying on Crystal Violet staining solubilisation and quantification. Both methods captured in a similar way and correctly the increase of biofilm formation over time. Surface-based quantification displayed a better amplitude measurement and therefore a more accurate quantification.

Supplementary Table 1

Strains	Velocity ($\mu m.s^{-1}$ ± sem)	Minimum ($\mu m.s^{-1}$)	Maximum ($\mu m.s^{-1}$)
Diguanylate cyclase A mutant (dgcA::Km)	5.67 ± 0.32	1.16	30.15
Aldoketoreductase A mutant (akrA::Km)	2.75± 0.14	0.93	17.51
Wild type	2.89 ± 0.17	1.02	18.34
Phosphodiesterase A mutant (pdeA::Km)	2.06 ± 0.11	0.89	13.31
Phosphodiesterase B mutant (pdeB::Km)	1.99 ± 0.05	0.95	5.56

Supplementary Table 2

Strains	C-di-GMP (ng /mg of dry weight ± SD)
Diguanylate cyclase A mutant (dgcA::Km)	4.81 ± 1.42
Complemented Diguanylate cyclase A mutant (C-dgcA)	14.71 ± 3.70
Aldoketoreductase A mutant (akrA::Km)	10.87 ± 1.54
Complemented Aldoketoreductase A mutant (C-akrA)	11.93 ± 3.52
Wild type	11.50 ± 2,74
Phosphodiesterase A mutant (pdeA::Km)	38.86 ± 7.22
Complemented Phosphodiesterase A mutant (C-pdeA)	3.58 ± 2.56
Phosphodiesterase B mutant (pdeB::Km)	27.93 ± 6.43
Complemented Phosphodiesterase b mutant (C-pdeB)	11.85 ± 3.89

Supplementary Table 3: Bacterial strains used in this study

Leptospira interrogans, serogroup Pyrogenes, serovar Manilae, strain L495
Leptospira interrogans, serogroup Pyrogenes, serovar Manilae, strain L495, Mutants dgcA::km, pMAORI : empty
Leptospira interrogans, serogroup Pyrogenes, serovar Manilae, strain L495, Mutants C-dgcA, pMAORI : LMANV2_v2_150005
Leptospira interrogans, serogroup Pyrogenes, serovar Manilae, strain L495, Mutants akrA::km, pMAORI : empty
Leptospira interrogans, serogroup Pyrogenes, serovar Manilae, strain L495, Mutants C-akrA, pMAORI : LMANV2_v2_50029
Leptospira interrogans, serogroup Pyrogenes, serovar Manilae, strain L495, Mutants pdeB::km, pMAORI : empty
Leptospira interrogans, serogroup Pyrogenes, serovar Manilae, strain L495, Mutants C-pdeB, pMAORI : LMANV2_v2_270021
Leptospira interrogans, serogroup Pyrogenes, serovar Manilae, strain L495, Mutants pdeA::km, pMAORI : empty
Leptospira interrogans, serogroup Pyrogenes, serovar Manilae, strain L495, Mutants C-pdeA, pMAORI : LMANV2_v2_90001
Leptospira interrogans, serogroup Icterohaemorrhagiae, serovar copenhageni, strain Fiocruz L1-130
Leptospira borgpetersenii, serougroup Ballum, serovar Ballum, strain B3-13S
Leptospira barantonii, strain FH4-C-A1
Leptospira ellisii, strain ATI7-C-A5
Leptospira adleri, strain FH2-B-C1
Leptospira kmetyi, strain JW3-C-A1
Leptospira hartskeerlii, strain MCA1-C-A
Leptospira neocaledonica, strain ES4-C-A1
Leptospira haakeii, strain ATI7-C-A2
Leptospira wolffii, strain FH2-C-A2
Leptospira perolatii, strain FH1-B-B1
Leptospira harrisiae strain FH2-B-A1
Leptospira levettii, strain MCA2-B-A1
Leptospira brenneri, strain JW2-C-A2
Leptospira meyeri, strain ATI2-C-A2
Leptospira biflexa, strain Patoc-1

Legends Supplementary Movies

Supplementary Movie 1.

Time-lapse imaging of *Leptospira* biofilm formation. Kinetic of biofilm formation captured through phase contrast images acquisition using a compact cell incubator and monitoring system (BioStation IM-Q). Time-lapse imaging started 12h after seeding under static condition at 30°C and 95% of humidity. Phase contrast images were acquired every 30 min over a period of 9 days using a 20X objective (field of view: 413*311µm).

Supplementary Movie 2.

Time-lapse sequence illustrating the synchronized and collective contraction process that lead to the formation of “ring like” structures. Phase contrast images were acquired every 30 min over a period of 6 hours using a 20X objective (field of view: 311*311µm). White arrows highlight biofilm aggregates of interest. Red arrows highlight spreading biofilm aggregates and green arrows indicate aggregates forming the “ring-like” structure.

Supplementary File 6: MALDI-ToF analysis of the biofilm ECM, methods & results

Materials and Methods

MALDI-TOF analysis

Twenty mL of a 10^6 bacteria mL^{-1} suspension were inoculated into a 50 mL untreated tissue culture flask (25cm² Rectangular Canted Neck Tissue Culture Flask, 353014, Falcon) and incubated at 30°C under static conditions. After three weeks, supernatant containing planktonic bacteria was gently removed. PBS was added and flasks were incubated at 30°C for 24h in order to rinse the biofilm from EMJH components that could interfere with Matrix Assisted Laser Desorption Ionisation - Time of Flight (MALDI-TOF) experiments (i.e: Tween 80). Biofilm were then collected and lyophilized prior to MALDI-TOF analysis. Five mg of biofilm (dry mass) was solubilized in deionized water then subjected to multiple sonication, 56°C incubation, and vortex homogenization cycles, until no residual biofilm particles were observable. Samples were mixed (1:1, v/v) with alpha-cyano hydroxycinnamic acid (CHCA) (4mg/mL in 60:40 (v/v) acetonitrile/water) and spotted on a stainless steel MALDI probe. Mass spectrometry acquisition was performed on an AB Sciex MALDI-TOF/TOF 5800 system, on 500-4000 m/z range, in positive reflectron mode, using 5000 laser shots per spectrum in continuous stage motion. Accuracy better than 50ppm was reached with external calibration (using 13 calibration spots on the MALDI probe). MS/MS fragmentation was achieved using 1kV or 2kV methods, with or without collision gas, and with either 3 m/z or 100 m/z precursor selection windows.

Results.

A MALDI-TOF analysis of the matrix showed mass peaks at 3000-3500 m/z, and a mass difference between the peaks of 14.9 m/z (supplemental file S1 figure 1A, B). A correspondence with known adducts could not be established (supplemental file S1 figure 1C).

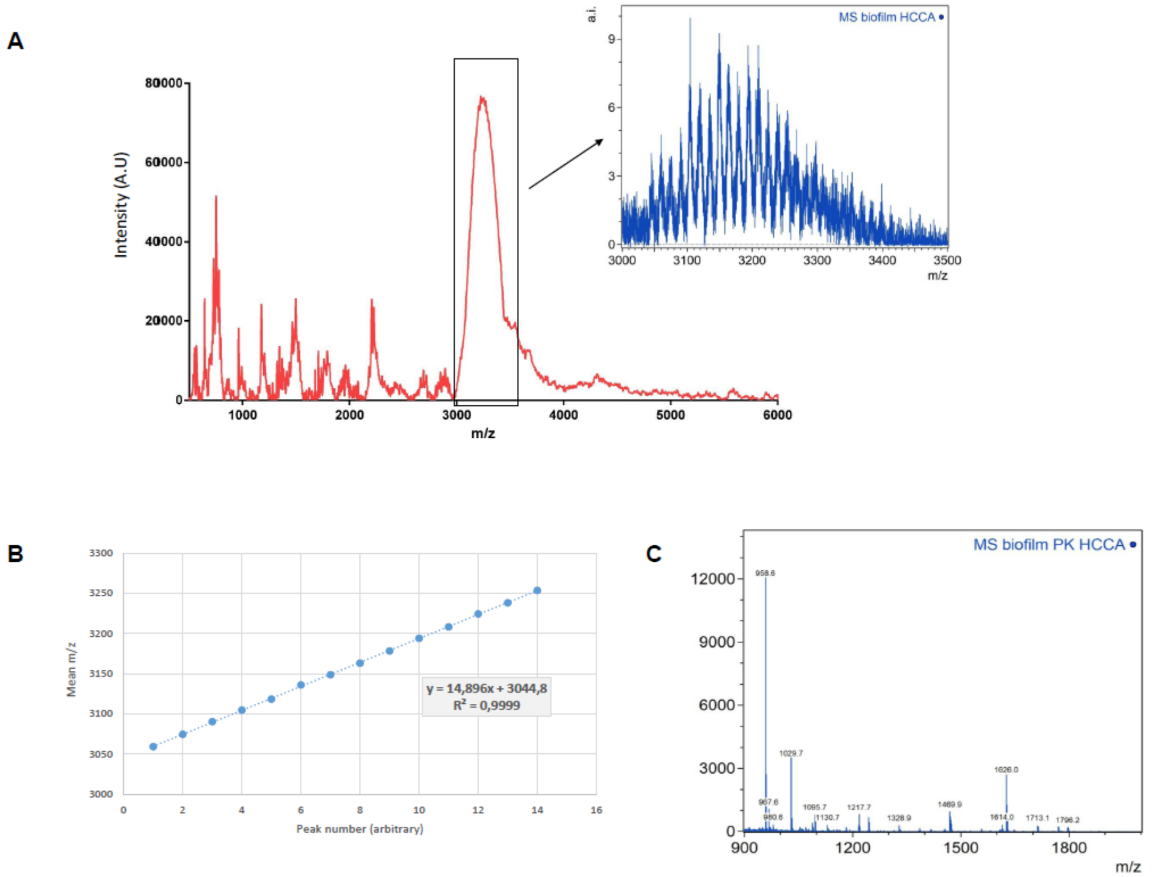

Supplementary file 6.

(A) MALDI-TOF MS analysis of biofilm composition showing the intensity of the signal as a function of the ratio m/z. The inset shows a magnification of the peak between 3,000 and 3,500 m/z. (B) Graph showing m/z of the biofilm between 3,050 and 3,250 m/z as a function of the number of the peak. The equation of the linear regression indicates that peaks are separated by 14.9 m/z. (C) MALDI-TOF MS analysis of biofilm after incubation with proteinase K (PK), major peaks are PK autolysis products.
